# Modeling Classical Swine Fever Outbreak-Related Outcomes

**DOI:** 10.3389/fvets.2016.00007

**Published:** 2016-02-03

**Authors:** Shankar Yadav, Nicole J. Olynk Widmar, Hsin-Yi Weng

**Affiliations:** ^1^Department of Comparative Pathobiology, Purdue University, West Lafayette, IN, USA; ^2^Department of Agricultural Economics, Purdue University, West Lafayette, IN, USA

**Keywords:** classical swine fever, disease modeling, epidemic, disease outbreak control, swine, pigs

## Abstract

The study was carried out to estimate classical swine fever (CSF) outbreak-related outcomes, such as epidemic duration and number of infected, vaccinated, and depopulated premises, using defined most likely CSF outbreak scenarios. Risk metrics were established using empirical data to select the most likely CSF outbreak scenarios in Indiana. These scenarios were simulated using a stochastic between-premises disease spread model to estimate outbreak-related outcomes. A total of 19 single-site (i.e., with one index premises at the onset of an outbreak) and 15 multiple-site (i.e., with more than one index premises at the onset of an outbreak) outbreak scenarios of CSF were selected using the risk metrics. The number of index premises in the multiple-site outbreak scenarios ranged from 4 to 32. The multiple-site outbreak scenarios were further classified into clustered (*N* = 6) and non-clustered (*N* = 9) groups. The estimated median (5th, 95th percentiles) epidemic duration (days) was 224 (24, 343) in the single-site and was 190 (157, 251) and 210 (167, 302) in the clustered and non-clustered multiple-site outbreak scenarios, respectively. The median (5th, 95th percentiles) number of infected premises was 323 (0, 488) in the single-site outbreak scenarios and was 529 (395, 662) and 465 (295, 640) in the clustered and non-clustered multiple-site outbreak scenarios, respectively. Both the number and spatial distributions of the index premises affected the outcome estimates. The results also showed the importance of implementing vaccinations to accommodate depopulation in the CSF outbreak controls. The use of routinely collected surveillance data in the risk metrics and disease spread model allows end users to generate timely outbreak-related information based on the initial outbreak’s characteristics. Swine producers can use this information to make an informed decision on the management of swine operations and continuity of business, so that potential losses could be minimized during a CSF outbreak. Government authorities might use the information to make emergency preparedness plans for CSF outbreak control.

## Introduction

Classical swine fever (CSF) is a highly contagious viral disease found in domestic and wild pigs. Due to its severe economic and animal welfare consequences, CSF is classified as a reportable disease by the World Organisation for Animal Health (OIE). From 1997 to 1998, several European countries experienced CSF outbreaks (1). In the Netherlands, outbreaks continued for 401 days, and more than 11 million pigs were killed (2). The total economic losses in the Netherlands due to the outbreaks were estimated to be $2.3 billion (3), and 50% of the losses were attributed to the “welfare slaughter” of pigs (4). The term welfare slaughter is defined as the mass depopulation of healthy pigs due to the emergence of animal welfare concerns on swine premises (5). During the 1997–1998 CSF outbreaks in Spain, the Netherlands, and Germany, the welfare slaughter proportions were 87, 64, and 60%, respectively (1, 2).

Although CSF was eradicated from the United States in 1978, it is one of the most important foreign animal diseases (FADs) along with foot-and-mouth disease (FMD) and highly pathogenic avian influenza (6). Currently, many Asian, Latin American, and European countries are CSF-endemic regions (7). International trade, pork smuggling, and immigrant farm workers entering from endemic regions could be potential risks for CSF introduction in the United States (8). The over four million feral pigs that live in 39 states of the United States also pose a potential threat for CSF spread (9, 10). These feral pigs might serve as a means to spread CSF if they were accidentally infected by consuming contaminated feed or from contact with infected pigs from neighboring CSF-endemic countries.

A potential CSF outbreak would lead to an immediate international trade ban on pork and pork products that would last for at least 3 months in the affected areas (11). The United States swine industry, one of the world’s top pork exporters, might face huge economic consequences due to trade ban. In 2014, the United States swine industry was worth an estimated $6.67 billion in the export market (12), which might be seriously affected by a CSF outbreak. However, the actual economic impacts of CSF on the United States swine industry will be dependent upon temporal and spatial patterns as well as the extent of an outbreak. Because CSF is not present in the United States, epidemiological modeling and simulation studies can be applied to estimate the patterns and magnitude of a potential outbreak.

The published simulation studies on the spread of CSF were based on swine production in Belgium (13), Germany (14), the Netherlands (15), Denmark (16), and Spain (17). The modeling approaches and strategies used across these studies were similar in that the simulations of disease outbreaks were initiated from single swine premises. In reality, CSF outbreaks might occur at several locations simultaneously because of swine operations sharing the same import origins. These outbreaks would have different epidemiological, economic, and animal welfare consequences than previously reported. Furthermore, the epidemiological estimates derived from these models cannot be directly extrapolated to the United States due to differences in the European swine production system, potential initial outbreak characteristics, and control strategies. Therefore, we investigated the likely patterns of a CSF outbreak while considering the swine production system, the movement of pigs, and other attributes of CSF introduction in Indiana in the United States. The goal of this study was to provide a decision tool to the end users that could be easily updated with routinely collected data to generate timely evidence-based outbreak outcome estimates. The objective of this study was to estimate CSF outbreak-related outcomes, such as epidemic duration and number of infected, vaccinated, and depopulated premises, from the defined most likely CSF outbreak scenarios by using the swine premises data of Indiana.

## Materials and Methods

The study was conducted in two steps: (1) selecting the most likely CSF outbreak scenarios in Indiana and (2) simulating the selected CSF outbreak scenarios to estimate outbreak outcomes.

### Selecting the Most Likely CSF Outbreak Scenarios in Indiana

Both county- and premises-level risk metrics for defining CSF introduction scenarios in Indiana were established. The attributes that were associated with CSF introduction according to published literature were identified from different data sources, including the 2012 Indiana State Swine Premise Identification Database (USAHERDS), United States Census data, and the 2013 Indiana State Natural Resources Data (Table 1). USAHERDS included information on individual swine premises and their live pig import and export activities. Import data included shipments of live pigs coming into Indiana from other states of the United States or abroad, while the export data included the shipments of live pigs leaving Indiana. All of the import and export activities were listed with the date of veterinary inspection, shipment size, geolocations (latitude and longitude) of import origin, receiving swine premises, and shipment date. As the movement of pigs is well recognized as a potential risk for CSF introduction in a CSF-free area (8, 18), several import activity-related attributes were included in the risk metrics.

**Table 1:** **Summary of county- and premises-level attributes used for the selection of the most likely classical swine fever outbreak scenarios in Indiana, United States**.

Attribute	County level	Premises level

	Median (5th, 95th percentiles)	
Import frequency	15 (1, 122)	2 (1, 17)
International import frequency	8 (1, 33)	1 (1, 5)
Number of imported pigs	10,735 (2, 127,992)	750 (1, 19,960)
Number of import origins	6 (1, 30)	1 (1, 5)
Number of swine premises	91 (22, 238)
Type of swine premises	
IDEM	9 (1, 56)
NP	82 (20, 183)
Exhibition	1 (1, 1)
Operation types	
Nursery, nursery-to-finish	2 (1, 16)
Finish	4 (1, 27)
Farrow-to-finish	4 (1, 19)
Immigrant population	749 (101, 18,163)

All of the swine premises in Indiana were identified by a unique swine premises identification number, geolocation (latitude and longitude), herd size, and operation type. These characteristics of swine premises were important determinants for CSF introduction. For example, nursery operations have frequent movement of pigs, people, and vehicles due to a shorter operation cycle, which might put them at higher risk for CSF introduction than other swine operations. Furthermore, the aggregation of pigs from different swine premises during an exhibition event might increase the risk for CSF introduction and spread and was included in the risk metrics as an attribute (19, 20).

There are two types of premises listed in USAHERDS: premises permitted by the Indiana State Department of Environmental Management (IDEM) and non-permitted (NP) premises. In Indiana, it is mandatory to receive permission from IDEM to run swine operation if the herd size is ≥700. Unlike IDEM premises, the NP premises are not subject to mandatory government inspection. Therefore, permit type (i.e., IDEM/NP) was included as a metric attribute for CSF introduction (21).

The 2012 immigrant population and total population of Indiana by county (*N* = 92) were extracted from the American Community Survey Data website (http://factfinder2.census.gov/). Immigrant swine farm workers from CSF-endemic countries might be an important risk for CSF introduction in the United States (21). Because the exact statistics on immigrant workers were not available at county level, an index variable was computed using Eq. 1.

(1) $$\begin{array}{l} \text{Immigrant index for county }j\\ =({\text{Immigrant population}}_{j}/{\text{Total population}}_{j})\\ \;\times \;{\text{Total swine premises}}_{j} \end{array}$$

The Indiana State Natural Resources Data included the spatial distribution of Indiana’s feral pig population. Feral hogs are one of the reported sources for CSF outbreaks in Germany (10). In the United States, feral hogs are present in at least 39 states (including Indiana) with an estimated population size of more than four million (9). CSF might be introduced in a naive feral pig population through contaminated food scraps or other unknown sources, which might spread the disease into Indiana. The data on Indiana’s feral hog population distribution were routinely collected and were included as an attribute in the risk metric.

These data were analyzed, and the distribution of each metric attribute was examined to determine the cutoff values for categorization by nature gaps in the data or percentiles. Corresponding weights were assigned based on the importance of the attributes to CSF introduction with a larger weight representing a higher risk. The county-level risk metric (Table 2) was used to score each of the Indiana’s counties based on the data for their risk of CSF introduction. The counties were then ranked by the summed score across all the metric attributes. The top 10 counties with the highest scores were identified and divided into 2 groups: top 1–5 and top 6–10. Using a similar algorithm, the swine premises in the top 1–5 and top 6–10 counties were ranked separately based on the premises-level metrics (Table 3). The top-ranked swine premises with the highest weights were selected.

**Table 2:** **The scoring system for the county-level risk metrics for selecting the most likely classical swine fever outbreak scenarios in Indiana, United States**.

Attribute	Weight range	Category	Weight
Import frequency	0–5	No import	0
		≤20th percentile	1
		20th to 40th percentile	2
		40th to 60th percentile	3
		60th to 80 percentile	4
		>80th percentile	5
International import frequency	0–5	No import	0
		≤20th percentile	1
		20th to 40th percentile	2
		40th to 60th percentile	3
		60th to 80th percentile	4
		>80th	5
Number of imported pigs	0–3	No import	0
		≤50,000	1
		50,001–100,000	2
		>100,000	3
Number of import origins	0–3	No import	0
		≤50th percentile	1
		50th to 75th percentile	2
		>75th percentile	3
Number of swine premises	0–5	No premises	0
		≤20th percentile	1
		20th to 40th percentile	2
		40th to 60th percentile	3
		60th to 80th percentile	4
		>80th percentile	5
Number of non-permitted swine premises	0–5	No premises	0
		≤20th percentile	1
		20th to 40th percentile	2
		40th to 60th percentile	3
		60th to 80th percentile	4
		>80th percentile	5
Exhibition swine premises	0 or 2	Absence	0
		Presence	2
Number of nursery and nursery-to-finish premises	0–2	Absence	0
		≤15	1
		>15	2
Immigrant population index	0–2	≤2	0
		2–7	1
		>7	2
Feral hogs	0 or 3	Absence	0
		Presence	3

**Table 3:** **The scoring system for the premises-level risk metrics for selecting the most likely classical swine fever outbreak scenarios in Indiana, United States**.

Attribute	Weight range	Category	Weight
Import frequency	0–5	No import	0
		≤25th percentile	1
		25th to 75th percentile	3
		>75th percentile	5
International import frequency	0–2	No import	0
		≤4	1
		>4	2
Number of imported pigs	0–3	No import	0
		≤25th percentile	1
		25th to 75th percentile	2
		>75th percentile	3
Number of import origins	0–5	No import	0
		≤4	2
		>4	5
Type of swine premises	0 or 3	IDEM	0
		NP	3
Operation types	0 or 2	Finish, farrow-to-finish	0
		Nursery, nursery-to-finish	2

After selecting the swine premises with the highest risk of CSF introduction using the risk metrics, the next step was to define the most likely CSF outbreak scenarios in Indiana. Two types of CSF outbreak scenarios were considered: single and multiple sites. These two types of CSF outbreak scenarios were different in the number of index premises (i.e., initially infected premises) at the onset of an outbreak. Only one index premises was present in a single-site outbreak scenario, while more than one index premises were simultaneously present in a multiple-site outbreak scenario. Each swine premises selected from the risk metrics represented the location of index premises for a single-site outbreak scenario. Next, the index premises’ import origin for each single-site outbreak scenario was reviewed to identify other swine premises in Indiana that imported live pigs from the same source premises. These secondarily identified premises and the originally selected index premises for the single-site outbreak scenarios together comprised a multiple-site outbreak scenario. The process was repeated for each premises selected from the risk metrics to define different multiple-site outbreak scenarios.

USAHERDS was used to map the distribution of index premises and control zones for the selected single-site and multiple-site outbreak scenarios using ArcGIS (version 10.3, ESRI, Redlands, CA, USA). The CSF control zones were plotted for each swine premises using the geoprocessing tools of ArcGIS according to the USDA CSF response plan (6). An infected zone with a 3-km radius and a movement restriction zone (also called buffer zone) that was 7 km away from the infected zone’s perimeter were plotted. The qualitative (i.e., the mapping of index premises) and quantitative (i.e., the number of premises in control zones) data were extracted for all index premises. Furthermore, the spatial distribution of index premises was used to classify the selected multiple-site outbreak scenarios into clustered and non-clustered groups. The classification was based on the number of index premises and the distance among them. To be classified as a clustered scenario, a scenario must have more than 10 index premises, and the majority of index premises were within 20 km from each other. Therefore, if two control zones (each with a 10-km radius) overlapped, it indicates that the two index premises were located within 20 km from one another.

Sensitivity analyses for the risk metrics were performed on the operation type, number of pigs imported (county level) and international import frequency, and the number of import origins (premises level). These attributes were selected for sensitivity analysis because there were no obvious breaking points in the data to define low versus high risk. Different cutoff values were used to evaluate their impacts on the risk metrics. An attribute was considered to be sensitive if the change in its cutoff values resulted in retaining <70% of the same selection of the top-ranked counties and swine premises.

### Simulation of the CSF Spread

North American Animal Disease Spread Model (NAADSM) software PC version 4.0.13 (22) was used to simulate the selected CSF outbreak scenarios. NAADSM is a stochastic, temporal, and spatial state transition disease spread model. All Indiana swine premises registered in USAHERDS were included in the model simulations. It was assumed that a swine premises was infected if any pigs on the premises were infected. The simulations started with the index premises for each of the selected CSF outbreak scenarios (labeled as the infected) on day 0, whereas the rest premises were labeled as susceptible. Each of the selected single-site and multiple-site outbreak scenarios were simulated separately for 500 iterations. The model input parameters are listed in Table 4.

**Table 4:** **Premises-level model input parameters and the probability distributions used for the simulation of classical swine fever spread in Indiana, United States**.

Parameters	Probability distribution	Reference
**DISEASE PARAMETERS**		
Latent period (days)	Poisson (4)	(23)
Infectious sub-clinical period (days)	Poisson (6)	(24)
Infectious clinical period (days)	Poisson (21)	(17)
**DIRECT CONTACT SPREAD**		
Mean baseline direct contact rate (recipient units/day)	0.186	(25, 26)
Probability of infection transfer	0.277	(27)
Distance distribution of recipient units (km)	Triangle (1, 60, 120)	Empirical data
**INDIRECT CONTACT SPREAD**		
Mean baseline indirect contact rate (recipient units/day)	0.3	(14)
Probability of infection transfer	0.048	(27)
Distance distribution of recipient units (km)	Triangle (1, 60, 120)	Empirical data
**LOCAL SPREAD**		
Distance between two premises (km)	1	(28)
Daily probability of local areas spread of CSF between two premises	0.00001	(29)
**CONTROL ZONES**		
Movement restriction zone radius (km)	7	(6)
Infected zone radius (km)	3	(6)
**DEPOPULATION FOR CSF CONTROL**		
Delay before implementing depopulation program (days)	2	(25)
Depopulation ring radius (km)	3	(6)
**VACCINATION**		
Number of infected units to be investigated before vaccinations	5	(25)
Vaccination immune period (days)	Triangle (150, 240, 365)	(30)
Delay in immunity (days)	7	(30)
Radius of vaccination ring (km)	3	(6)

Simulations were run at the level of swine premises with a time step of 1 day. Three modes of CSF transmission (direct contact, indirect contact, and local spread) were used to model disease spread. Direct contact was defined as the spread of CSF from an infected premises to a susceptible premises through the shipment of pigs, whereas indirect contact was through movement of vehicles, people, and equipment. A local spread was defined as the spread of CSF from an infected premises to a susceptible premises within a 1-km radius by unknown mechanisms; possibly by insects or reptiles (22, 28, 31). For direct and indirect contact, probability distributions were assigned for three parameters: contact rate, probability of infection transfer due to a contact, and distance distribution of recipient premises. Estimates for direct and indirect contact rates were obtained from published studies (14, 17, 25). The probability of infection transfer due to a direct or an indirect contact was adopted from the estimate derived from the 1997–1998 CSF outbreaks in the Netherlands (27). In NAADSM, the distance matrix among swine premises was computed using their geolocations. During a simulation, the model algorithm selected recipient susceptible swine premises for a direct or indirect contact based on their distance from an infected premises. The closer susceptible swine premises had a higher probability of being infected than premises that were further away. The probability of disease transfer by local spread also gradually decreased with an increase in the distance from an infected premises (22, 31). Details on the three modes of disease transmission implemented in NAADSM can be found in Table 4 and other references (25, 28, 31).

Different outbreak control measures were allowed in NAADSM. The implementation of movement restriction, vaccination, and the depopulation of infected and vaccinated premises in the model was based on the CSF control strategies of the United States (6, 25). Due to the effects of movement restriction, the direct contact and indirect contact rates were reduced to 15 and 30% of their baseline by the seventh day, respectively. These reductions stayed constant by the 30th day in single-site outbreak scenarios. The reductions of contact rates reflect the compliance rate of movement restriction. For example, a reduction of 15% corresponds to a compliance rate of movement restriction of 85%. The compliance rate of movement restriction was obtained from published studies (23, 32). Compliances with movement restriction were assumed to be lower in multiple-site outbreak scenarios due to potential illegal movements in the wider geographical areas. Therefore, the direct and indirect contact rates were assumed to be reduced to 25 and 50% of their baseline at the 60th day onward in multiple-site outbreak scenarios. Movement restriction does not affect local spread of CSF in NAADSM. Vaccination (live attenuated) was implemented in the model for all swine premises in the infected zone (a 3-km radius of the infected premises) so that disease spread could be reduced, and depopulation capacity could be accommodated (6, 33, 34). All the infected and vaccinated swine premises were set to be depopulated during the CSF control. The depopulation capacity was modeled to gradually increase from 0 to 10 premises/day by the 7th day and 15 premises/day by the 15th day onward (25). The detailed algorithms of NAADSM can be found elsewhere (22, 31).

The major outcomes of the study were the median (5th, 95th percentiles) epidemic duration and the number of premises being infected, depopulated, and vaccinated. All iteration results were combined across the outbreak scenarios within single-site and multiple-site outbreaks when computing the outputs.

The direct and indirect contact rates and probability of infection transfer due to contact were chosen for the sensitivity analyses of the CSF spread model due to their inconsistent use in published studies (14, 16, 17, 35). A 25% change in the values of selected input parameters was used in the analyses to evaluate their effects on the estimates of epidemic duration and the number of infected premises. A parameter was considered sensitive if the 25% change in its values led to a change in the median epidemic duration by 10 days and a median percent of infected premises by 15% from their baseline estimates. The selection of thresholds for the sensitivity analysis was subjective. However, similar thresholds were used for sensitivity analyses in other simulation studies using NAADSM (25).

## Results

There were 8,631 (IDEM: 1,381, NP: 7,250) swine premises, 38 exhibition swine premises, 11 dealers, and 44 pig collection points registered in 2012 in Indiana. In total, 3,145 import shipments of live pigs were completed. A total of 365 swine premises outside of Indiana sent shipments of pigs to 765 swine premises in Indiana. The median numbers of pigs imported into Indiana in 2012 were 1,000 at the premises level and 10,571 at the county level. The import shipment size ranged from 1 to 11,175 (median: 1,000).

### The Most Likely CSF Outbreak Scenarios

Nineteen single-site and 15 multiple-site CSF outbreak scenarios were selected using the risk metrics. The number of index premises in the multiple-site outbreak scenarios ranged from 4 to 32. None of the selected attributes in the risk metrics were sensitive as more than 70% of the 10 top-ranked counties and swine premises remained in the sensitivity analyses.

**Figure 1:**
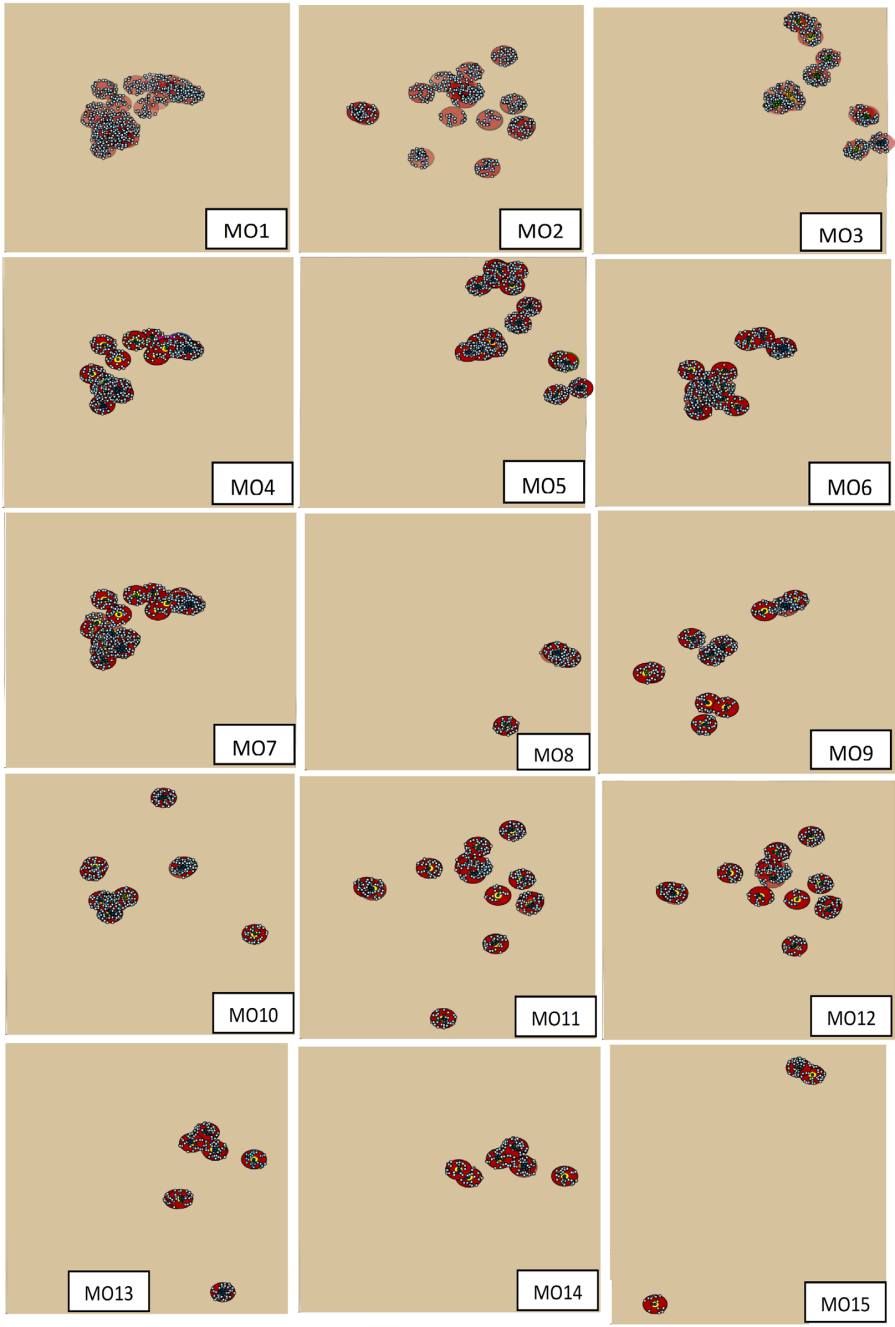
**The distribution of index swine premises and the outbreak control zones for the 15 most likely multiple-site classical swine fever outbreak scenarios (MO1 to MO15)**. The dots are the swine premises and the circles are the control zones.

Control zones were mapped for each of the 254 index premises in the selected outbreak scenarios. The spatial distribution of the index premises of multiple-site outbreaks and their control zones are present in Figure 1. For confidentiality, the distribution of single-site outbreak scenarios is not present. Among the multiple-site outbreak scenarios, six scenarios (MO1, MO2, MO4, MO5, MO6, and MO7, where “MO” represents the multiple-site outbreak scenarios, and the digit represents their ranking) were classified as clustered, while the rest were classified as non-clustered based on the criteria previously described (Figure 1). The number of index premises in the clustered scenarios ranged from 19 to 32, and ranged from 4 to 19 in the non-clustered scenarios. The median (5th, 95th percentiles) number of swine premises in the infected zones in the single-site and multiple-site outbreak scenarios was 6 (1, 10) and 64 (40, 90), respectively. The median (5th, 95th percentiles) number of swine premises in the movement restriction zones was 90 (28, 170) and 570 (150, 770) for the single-site and multiple-site outbreak scenarios, respectively.

### CSF Spread Outcomes

**Figure 2:**
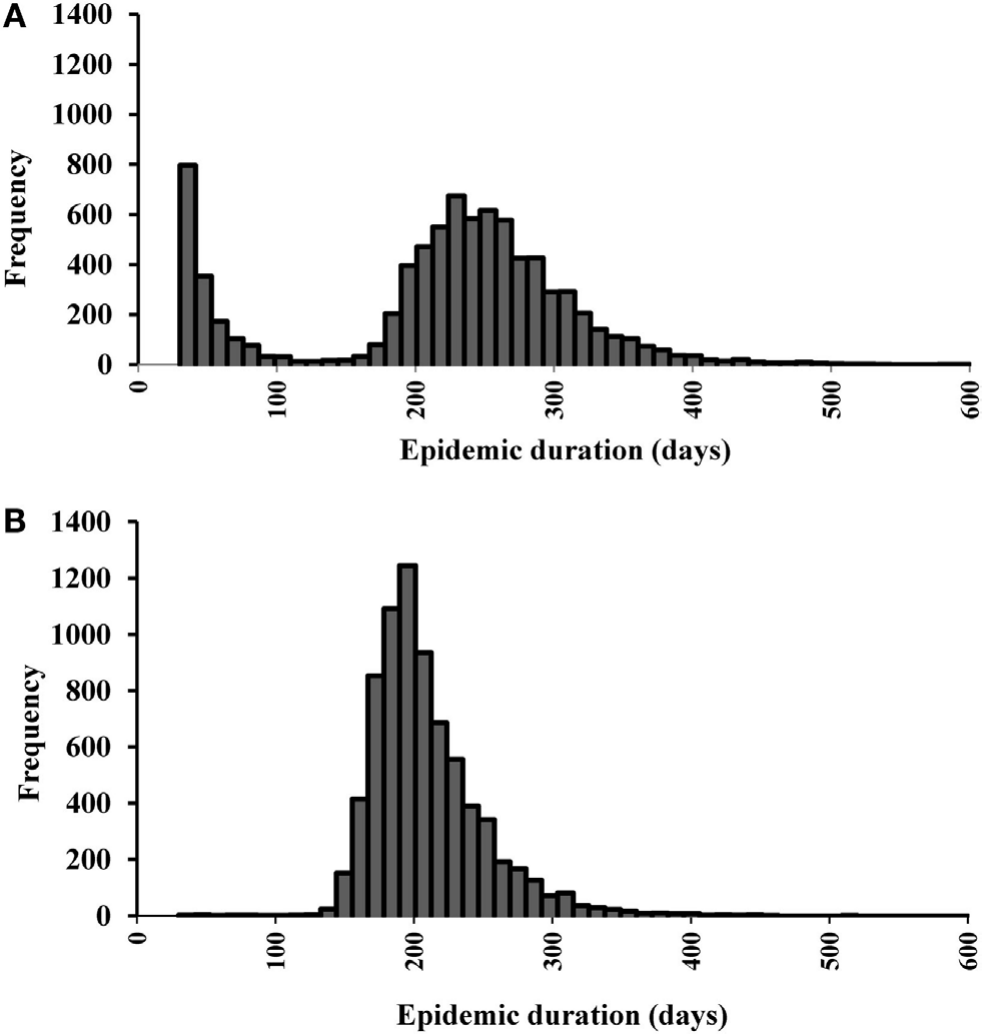
**Histograms of the epidemic duration estimates for (A) 19 most likely single-site classical swine fever outbreak scenarios (*N* = 9,500 iterations)**. **(B)** **15 most likely multiple-site outbreak scenarios (*N* = 7,500 iterations)**.

**Figure 3:**
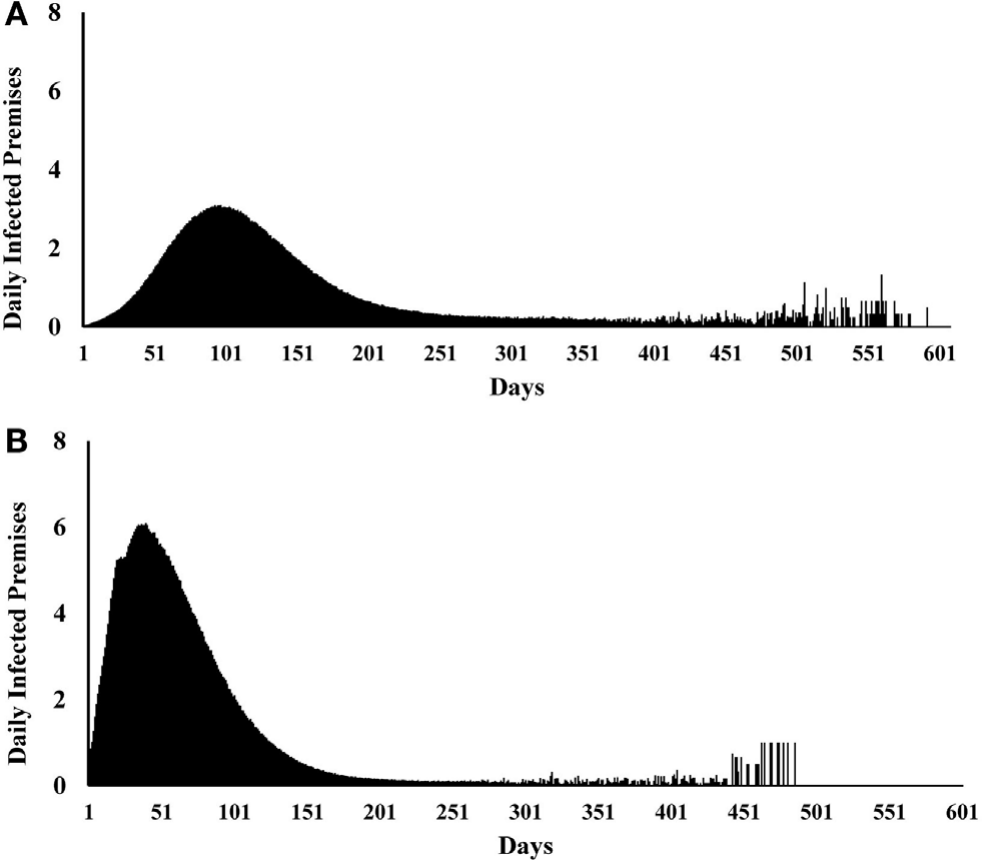
**Epidemic curves for (A) 19 most likely single-site classical swine fever outbreak scenarios (*N* = 9,500 iterations)**. **(B)** **15 most likely multiple-site outbreak scenarios (*N* = 7,500 iterations)**.

We initially estimated the outcomes separately for each of the 19 single-site and 15 multiple-site outbreak scenarios (data not shown). The results from the single-site outbreak scenario simulations were similar, whereas the results from the multiple-site outbreak scenario simulations varied based on the spatial patterns of the index premises (i.e., clustered versus non-clustered). Overall, we found that the local spread (58%) contributed the highest proportions of CSF transmission followed by direct (33%) and indirect (8%) contact. The estimated median (5th, 95th percentiles) epidemic duration (days) was 224 (24, 343) in the single-site and 201 (161, 285) in the multiple-site outbreak scenarios (Figures 2A,B). The median number of days to peak epidemic was 97 with the number of newly infected premises of 3.1 at the peak in the single-site outbreak scenarios (Figure 3A). The median number of days to peak epidemic was 40 with the number of newly infected premises of 6.8 at the peak in the multiple-site outbreak scenarios (Figure 3B). Among the multiple-site outbreak scenarios, the median (5th, 95th percentiles) epidemic duration was 190 (157, 251) in the clustered scenarios (estimated from 3,000 iterations) and 210 (167, 302) in the non-clustered scenarios (estimated from 4,500 iterations). A shorter time to peak epidemic (46 versus 33 days) and a larger number of newly infected premises at the peak (7.9 versus 5.2) was found in the clustered scenarios compared to the non-clustered scenarios.

**Figure 4:**
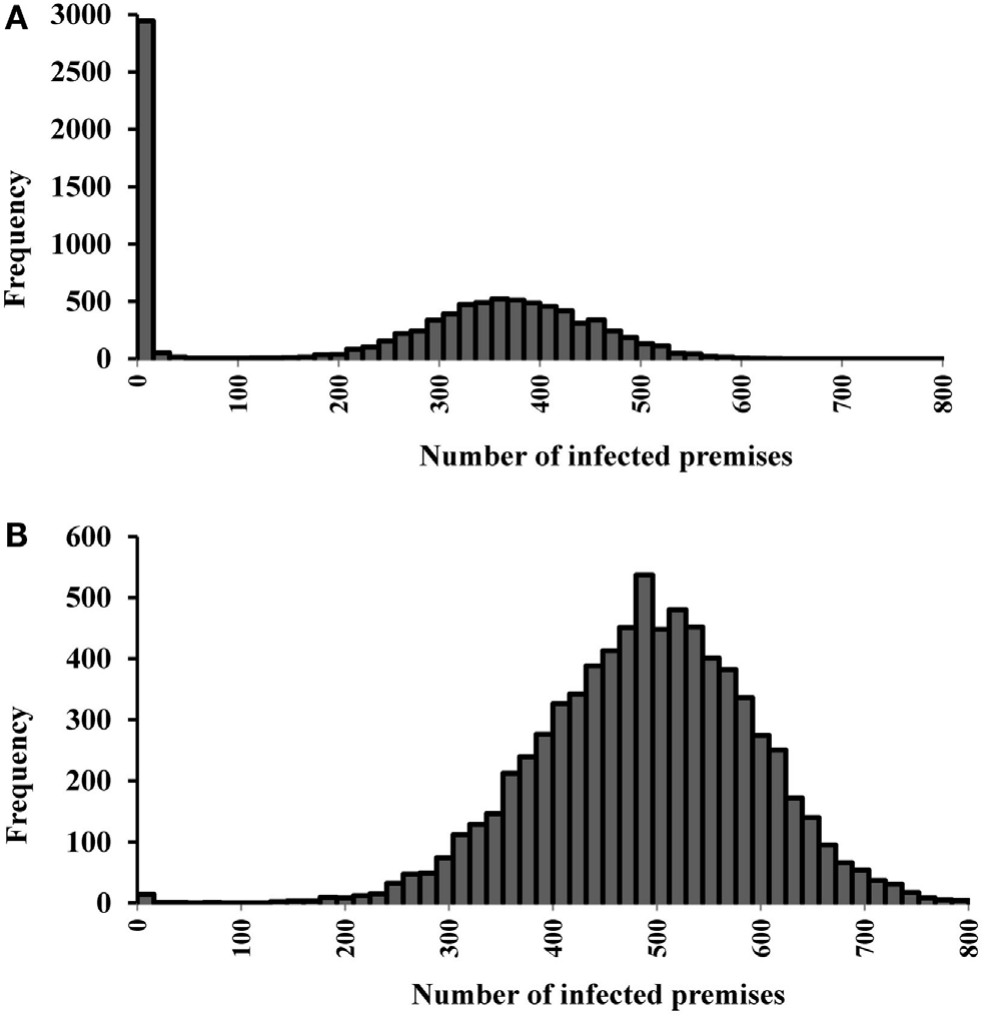
**Histograms of the number of infected premises estimates for (A) 19 most likely single-site classical swine fever outbreak scenarios (*N* = 9,500 iterations)**. **(B)** **15 most likely multiple-site outbreak scenarios (*N* = 7,500 iterations)**.

The median (5th, 95th percentiles) numbers of infected, vaccinated, and depopulated premises in the single-site and multiple-site outbreaks are present in Table 5. The distribution of number of infected premises is also present in the histograms (Figures 4A,B). Based on our models, vaccinations could be completed within 2 days in both the single-site and multiple-site scenarios. However, mass depopulations took several days to complete. The median number of days (5th, 95th percentiles) for premises to be in a queue for mass depopulation was 3 (1, 16) in the single-site and 23 (2, 44) in the multiple-site outbreak scenarios. The median number of days for premises to be in a queue for mass depopulation was longer in the clustered scenarios than in the non-clustered scenarios (31 versus 14 days).

**Table 5:** **The median (5th, 95th percentiles) number of infected, vaccinated, and depopulated swine premises in the 19 most likely single-site classical swine fever outbreak scenarios (*N* = 9,500 iterations) and 6 clustered (*N* = 3,000 iterations) and 9 non-clustered (*N* = 4,500 iterations) multiple-site outbreak scenarios**.

Outcome	Single-site outbreak	Multiple-site outbreak	
		Clustered	Non-clustered
Infected premises	323 (0, 488)	529 (395, 662)	465 (295, 640)
Vaccinated premises	405 (0, 1,621)	2,131 (1,526, 2,556)	1,655 (360, 5,475)
Depopulated premises	1,526 (5, 2,198)	2,659 (1,927, 3,211)	1,987 (657, 2,925)

The indirect contact rate and probability of infection transfer due to indirect contact were not sensitive in any of the scenarios. The epidemic duration estimates for the multiple-site outbreak scenarios were not affected by changes in the values of selected input parameters. However, epidemic duration estimates for the single-site outbreak scenarios were significantly reduced due to a 25% reduction in the direct contact rate and the probability of infection transfer due to direct contact. A 25% change in these input parameters also led to a significant change in the estimates of the number of infected premises in both single-site and multiple-site outbreak scenarios.

## Discussion

In this study, risk metrics were developed to select the most likely CSF outbreak scenarios for simulations using a disease spread model to estimate the magnitude and patterns of CSF outbreaks in Indiana in the United States, respectively. The goal of this study was to provide end users with an empirically supported decision tool that could be easily updated with routinely collected data to generate timely evidence-based estimates of CSF outbreak-related outcomes.

In the risk metrics for selecting the most likely CSF outbreak scenarios in Indiana, attributes for CSF introduction were identified, and a scoring system was developed based on the best available evidence compiled from empirical data, research literature, and expert opinion. A similar data collection approach was also used to obtain the data for the input parameters necessary to simulate CSF disease spread using NAADSM. The developed models and documented information would serve as a platform to assist in generating timely CSF outbreak-related outcomes at the onset of an outbreak.

Differences in the outbreak-related outcomes are evident in the historical CSF outbreaks. For example, the epidemic durations of the 1997–1998 CSF outbreaks in Germany, the Netherlands, and Spain were 123, 401, and 417 days, respectively (1). These differences might be attributed to the differences in the swine production system, the number of index premises at the onset of an outbreak and their spatial distribution, and the outbreak control strategies in the three countries. In this study, we defined different most likely CSF outbreak patterns (e.g., single-site, clustered multiple-site, and non-clustered multiple-site outbreaks) using risk metrics and investigated their effects on outbreak-related outcome estimates. The median epidemic duration estimates were longer for the single-site outbreak scenarios compared to the multiple-site outbreak scenarios (Figures 2A,B). However, a bimodal distribution was observed in the simulations of the single-site outbreak scenarios (Figure 2A). Out of the 9,500 iterations simulated in the single-site outbreak scenarios, 18% did not initiate an epidemic (Figure 4A) and 30% of iterations had epidemic durations of <100 days (Figure 2A). By contrast, all simulated iterations (7,500) in the multiple-site outbreaks resulted in an epidemic (Figure 4B), and only 0.15% of iterations had epidemic durations of <100 days (Figure 2B). The number of daily newly infected premises peaked at 3.1 in the single-site outbreak scenarios compared to a peak at 6.8 in the multiple-site outbreak scenarios (Figures 3A,B). A high number of daily newly infected premises in the multiple-site outbreak scenarios might have led to an insufficient number of susceptible premises in the population for the epidemic to continue, which resulted in an approximately 195-day mode of epidemic duration compared to the second mode that occurred at approximately 240 days in the single-site outbreaks (Figures 2A,B). These differences in the epidemic patterns between the single-site and multiple-site outbreak scenarios might explain the observation of a greater variation in the estimates of epidemic duration in the single-site outbreaks (i.e., a wider 5th and 95th percentile interval). Our estimates of the time to peak epidemic (97 days) and the number of peak daily infected premises (3.1) for the single-site outbreak scenarios were similar to the figures (98 days and 3.5, respectively) reported in the 1997–1998 CSF outbreaks in the Netherlands (32).

Our inclusion of the multiple-site outbreak scenarios allowed us to evaluate the impacts of the number of index premises (a range from 4 to 34) and their spatial distributions (clustered versus non-clustered) on the estimates of epidemic duration. The epidemics initiated with clustered index premises and with more index premises resulted in relatively shorter epidemic durations. For example, the estimated median epidemic duration was 174 in MO1 (the number of index premises was 32) and 253 in MO15 (the number of index premises was 4). Furthermore, for MO6 (clustered) and MO3 (non-clustered), which had the same number of index premises, the estimated median epidemic duration was 195 and 216, respectively. This observation provides further support to the previous hypothesis that a high number of index premises and clustered index premises might lead to an insufficient number of susceptible premises in the population for an epidemic to continue, resulting in a shorter epidemic duration.

Our approach differs from other similar modeling studies of the spread of CSF, which include only one single-site outbreak scenario (14, 16, 17, 36). The median epidemic durations estimated for single-site outbreak scenarios in this study were longer than that of an estimate in Spain (63 days) but were comparable to the estimates in Germany (200 days) and within the range of estimates in the Netherlands (100–373 days) (14, 17, 27). However, our results showed a bimodal distribution of the estimated epidemic durations, which indicated that using the median as a summary statistic might not be appropriate.

The median (5th, 95th percentile) proportion of infected premises in the single-site outbreak scenarios [3.7% (0, 5.5%)] in our study was comparable to other studies (14, 16, 17) and the 1997–1998 CSF outbreaks in the Netherlands (2%) (37). The multiple-site outbreak scenarios in our study resulted in a higher proportion of infected premises than the single-site outbreak scenarios.

The median proportion of depopulated premises (single-site outbreaks: 17.7%, multiple-site outbreaks: 26.3%) estimated in this study was higher than that of the 1997–1998 CSF outbreaks in the Netherlands (8%) (37). The difference might be explained by the incorporation of a bigger depopulation zone size in our study (3 km radius versus 1 km) (2). During a CSF outbreak, the availability of resources for vaccination and depopulation could greatly impact the success of the outbreak controls. In our study, 80% of the iterations simulated for the single-site outbreak scenarios completed depopulation within 1 week. In the multiple-site outbreak scenarios, only 20% were depopulated within 1 week, and 30% took more than 30 days for complete depopulation. Only 38% of the simulated iterations in the single-site outbreak scenarios and 9% in the multiple-site outbreak scenarios were depopulated within 48 h, which is the time required to depopulate infected and contact premises as defined in the USDA CSF response plan (6). Vaccination, in contrast to depopulation, was estimated to be completed within 2 days in all iterations for both single-site and multiple-site outbreak scenarios. Therefore, vaccination of swine premises in large geographical areas might be used to accommodate limited depopulation capacities to control the spread of disease given the modeled vaccination capacity in Indiana. These findings provide guidance on optimizing preparedness plans for CSF epidemics by implementing aggressive vaccination in outbreak controls (6).

The sensitivity analyses showed that the estimate of the number of infected premises was highly sensitive to a reduction in the direct contact rate and probability of CSF transmission due to direct contact, which indicates the importance of control strategies in reducing the epidemic size. For example, the implementation of movement restrictions would reduce the direct contact rate and strict biosecurity measures might help reduce the probability of CSF transmission after direct contact.

A caveat of the study is the lack of data on pig movement within Indiana, which might reduce the predictability of our risk metrics and the CSF spread model. We also used a higher degree of non-compliance in the CSF spread model for the multiple-site outbreak scenarios than the model for the single-site outbreak scenarios to account for larger geographical coverage. The difference in non-compliance rates would contribute to the differences in the outcome estimates observed between the single-site and multiple-site outbreak scenarios.

## Conclusion

The risk metrics and disease spread model were developed using available evidence collected from empirical data and published studies to estimate CSF outbreak-related outcomes. The risk metrics were used to select the most likely CSF outbreak scenarios in Indiana for disease simulations. The study demonstrates that the characteristics of an outbreak, whether single-site and multiple-site, affect the estimates of CSF outbreak-related outcomes. Our results also suggest that the implementation of an aggressive vaccination regime could accommodate limited depopulation capacity in controlling the spread of CSF. The estimated numbers of infected premises were sensitive to the direct contact rate and probability of disease transmission due to direct contact, which indicates the importance of control strategies in reducing direct contact. The outcome estimates generated from the study could assist swine producers in making informed decisions at the onset of a CSF outbreak to reduce potential economic losses. Our intent to develop the models using routinely collected data also allows other users to easily adapt and update the models to produce timely outcome estimates based on the characteristics of a CSF outbreak that they are facing.

## Author Contributions

SY: carried out study design, model development, simulations, data analysis, and manuscript writing; NW: contributed in data analysis and manuscript revision; HYW: PI of the project, contributed in study design, data analysis, and manuscript revision.

## Conflict of Interest Statement

The authors declare that the research was conducted in the absence of any commercial or financial relationships that could be construed as a potential conflict of interest.

## References

[1] Edwards S Fukusho A Lefevre PC Lipowski A Pejsak Z Roehe P Classical swine fever: the global situation. Vet Microbiol (2000) 73(2–3):103–19. 10.1016/S0378-1135(00)00138-3 10785321

[2] Elbers AR Stegeman A Moser H Ekker HM Smak JA Pluimers FH. The classical swine fever epidemic 1997-1998 in the Netherlands: descriptive epidemiology. Prev Vet Med (1999) 42(3–4):157–84. 10.1016/S0167-5877(99)00074-4 10619154

[3] Meuwissen MP Horst SH Huirne RB Dijkhuizen AA A model to estimate the financial consequences of classical swine fever outbreaks: principles and outcomes. Prev Vet Med (1999) 42(3–4):249–70. 10.1016/S0167-5877(99)00079-3 10619159

[4] Saatkamp HW Berentsen PB Horst HS. Economic aspects of the control of classical swine fever outbreaks in the European Union. Vet Microbiol (2000) 73(2–3):221–37. 10.1016/S0378-1135(00)00147-4 10785330

[5] Bargen LL Whiting TL. Time to critical overcrowding of Manitoba swine barns in the event of restriction on animal movement. Can Vet J (2002) 43(11):855–62. 12497962 PMC339757

[6] USDA. CSF outbreak response goals and strategy. In: Agriculture USDo, editor. Classical Swine Fever Response Plan: The Red Book. Riverdale, MD: Animal and plant health inspection service, veterinary services Chapter 4 (2013). p. 1–22.

[7] Classical Swine Fever Distribution Map [Internet]. World Animal Health Information Database (WAHID) (2015). Available from: http://www.oie.int/wahis_2/public/wahid.php

[8] Fevre EM Bronsvoort BM Hamilton KA Cleaveland S Animal movements and the spread of infectious diseases. Trends Microbiol (2006) 14(3):125–31. 10.1016/j.tim.2006.01.004 16460942 PMC7119069

[9] USDA. Feral/Wild Pigs: Potential Problems for Farmers and Hunters. Riverdale, MD: United States Department of Agriculture (USDA), Animal and Plant Health Inspection Service (APHIS) (2005).

[10] Penrith ML Vosloo W Mather C. Classical swine fever (hog cholera): review of aspects relevant to control. Transbound Emerg Dis (2011) 58(3):187–96. 10.1111/j.1865-1682.2011.01205.x 21303492

[11] World Organisation for Animal Health. Terrestrial animal health code. Volume II: Recommendations Applicable to OIE Listed Diseases and Other Diseases of Importance to International Trade. Paris: OIE (World Organisation for Animal Health) (2013). p. 385–666.

[12] NPC. National Pork Council (NPC) (2015). Available from: http://www.nppc.org/pork-facts/

[13] Ribbens S Dewulf J Koenen F Maes D de Kruif A. Evidence of indirect transmission of classical swine fever virus through contacts with people. Vet Rec (2007) 160(20):687–90. 10.1136/vr.160.20.687 17513834

[14] Karsten S Rave G Krieter J Monte Carlo simulation of classical swine fever epidemics and control. I. General concepts and description of the model. Vet Microbiol (2005) 108(3–4):187–98. 10.1016/j.vetmic.2005.04.009 15908147

[15] Nielen M Jalvingh AW Meuwissen MP Horst SH Dijkhuizen AA Spatial and stochastic simulation to evaluate the impact of events and control measures on the 1997-1998 classical swine fever epidemic in the Netherlands. II. Comparison of control strategies. Prev Vet Med (1999) 42(3–4):297–317. 10.1016/S0167-5877(99)00081-1 10619161

[16] Boklund A Toft N Alban L Uttenthal A. Comparing the epidemiological and economic effects of control strategies against classical swine fever in Denmark. Prev Vet Med (2009) 90(3–4):180–93. 10.1016/j.prevetmed.2009.04.008 19439381

[17] Martinez-Lopez B Ivorra B Ramos AM Sanchez-Vizcaino JM A novel spatial and stochastic model to evaluate the within- and between-farm transmission of classical swine fever virus. I. General concepts and description of the model. Vet Microbiol (2011) 147(3–4):300–9. 10.1016/j.vetmic.2010.07.009 20708351

[18] Elbers AR Stegeman JA de Jong MC. Factors associated with the introduction of classical swine fever virus into pig herds in the central area of the 1997/98 epidemic in the Netherlands. Vet Rec (2001) 149(13):377–82. 10.1136/vr.149.13.377 11601514

[19] Robinson SE Christley RM. Exploring the role of auction markets in cattle movements within Great Britain. Prev Vet Med (2007) 81(1–3):21–37. 10.1016/j.prevetmed.2007.04.011 17482296

[20] Bender JB Shulman SA. Reports of zoonotic disease outbreaks associated with animal exhibits and availability of recommendations for preventing zoonotic disease transmission from animals to people in such settings. J Am Vet Med Assoc (2004) 224(7):1105–9. 10.2460/javma.2004.224.1105 15074855

[21] CFSPH. Classical Swine Fever Ames: Center for Food Security and Public Health (2015). Available from: http://csf.cfsph.iastate.edu/index.php

[22] NAADSM, inventor. NAADSM Development Team 4.2. Free Program Distributed via the Internet (2013). Available from: http://www.naadsm.org

[23] Thulke HH Eisinger D Beer M. The role of movement restrictions and pre-emptive destruction in the emergency control strategy against CSF outbreaks in domestic pigs. Prev Vet Med (2011) 99(1):28–37. 10.1016/j.prevetmed.2011.01.002 21300412

[24] Klinkenberg D Nielen M Mourits MC de Jong MC. The effectiveness of classical swine fever surveillance programmes in the Netherlands. Prev Vet Med (2005) 67(1):19–37. 10.1016/j.prevetmed.2004.10.003 15698906

[25] McReynolds SW Sanderson MW Reeves A Hill AE. Modeling the impact of vaccination control strategies on a foot and mouth disease outbreak in the Central United States. Prev Vet Med (2014) 117(3–4):487–504. 10.1016/j.prevetmed.2014.10.005 25457133

[26] McReynolds SW Sanderson MW Reeves A Sinclair M Hill AE Salman M. Direct and indirect contact rates among livestock operations in Colorado and Kansas. JAVMA (2014) 244(9):1066–74. 10.2460/javma.244.9.1066 24739117

[27] Mangen MJ Nielen M Burrell AM. Simulated effect of pig-population density on epidemic size and choice of control strategy for classical swine fever epidemics in the Netherlands. Prev Vet Med (2002) 56(2):141–63. 10.1016/S0167-5877(02)00155-1 12450686

[28] Mintiens K Laevens H Dewulf J Boelaert F Verloo D Koenen F. Risk analysis of the spread of classical swine fever virus through “neighbourhood infections” for different regions in Belgium. Prev Vet Med (2003) 60(1):27–36. 10.1016/S0167-5877(03)00080-1 12900147

[29] Stegeman JA Elbers AR Boum A de Jong MC. Rate of inter-herd transmission of classical swine fever virus by different types of contact during the 1997-8 epidemic in the Netherlands. Epidemiol Infect (2002) 128(2):285–91. 10.1017/S0950268801006483 12002547 PMC2869822

[30] Blome S Meindl-Boehmer A Loeffen W Thuer B Moennig V. Assessment of classical swine fever diagnostics and vaccine performance. Rev Sci Tech (2006) 25(3):1025–38. 17361768

[31] Reeves A Hupalo R Calculating Probabilities of Local-Area and Airborne Disease Spread in NAADSM 4. Technical Paper 6. Fort Collins: NAADSM Development Team, Colorado State University (2012).

[32] Terpstra C de Smit AJ. The 1997/1998 epizootic of swine fever in the Netherlands: control strategies under a non-vaccination regimen. Vet Microbiol (2000) 77(1–2):3–15. 10.1016/S0378-1135(00)00252-2 11042396

[33] USDA APHIS. In: Agriculture USDo, editor. Foreign Animal Disease Framework Response Strategies: FAD Preparedness and Response Plan Manual 2-0. Riverdale, MD: Animal and Plant Health Inspection Service, Veterinary Services (2012).

[34] USDA APHIS. FMD outbreak response goals and strategy. In: Agriculture USDo, editor. Foot and Mouth Disease Response Plan: The Red Book. Chapter 4. Riverdale, MA: Animal and Plant Health Inspection Service, Veterinary Services (2012). p. 1–38.

[35] Jalvingh AW Nielen M Maurice H Stegeman AJ Elbers AR Dijkhuizen AA Spatial and stochastic simulation to evaluate the impact of events and control measures on the 1997-1998 classical swine fever epidemic in the Netherlands. I. Description of simulation model. Prev Vet Med (1999) 42(3–4):271–95. 10619160 10.1016/s0167-5877(99)00080-x

[36] Klinkenberg D Everts-van der Wind A Graat EA de Jong MC. Quantification of the effect of control strategies on classical swine fever epidemics. Math Biosci (2003) 186(2):145–73. 10.1016/j.mbs.2003.08.005 14583170

[37] Boender GJ Hengel Rvd Roermund HJWv Hagenaars T The influence of between-farm distance and farm size on the spread of classical swine fever during the 1997-1198 epidemic in the Netherlands. PLoS One (2014) 9(4):e95278 10.1371/journal.pone.0095278.t001 24748233 PMC3991596

